# Factors influencing institutional delivery and the role of accredited social health activist (ASHA): a secondary analysis of India human development survey 2012

**DOI:** 10.1186/s12884-020-03127-z

**Published:** 2020-08-05

**Authors:** Pooja L. Paul, Shanta Pandey

**Affiliations:** Boston College, School of Social Work, McGuinn Hall 204, 140 Commonwealth Avenue, Chestnut Hill, MA 02467 USA

**Keywords:** Accredited social health activist, Antenatal care, Community health workers, India, Delivery care

## Abstract

**Background:**

India has focused on incentivizing institutional delivery and introducing the ASHA worker as a key strategy to improve maternal health outcomes. We examined the determinants of institutional delivery and the role of the ASHA worker in shaping choice regarding place of delivery.

**Methods:**

We used data from the India Human Development Survey-II conducted in 2011–12, and extracted an analytic sample of women (*N* = 8711) who reported having at least one child since 2005. Logistic regression was used to examine influence of socio-demographic factors, frequency of antenatal care (ANC) contacts and exposure to ASHA worker on institutional delivery.

**Results:**

About 15% of the respondents had eight or more ANC contacts. The odds of having an institutional delivery were higher among those respondents who had 8 or more ANC contacts (OR = 3.39, *p* < 0.001, 95% CI: 2.26, 5.08), and those who had 4–7 ANC contacts (OR = 1.72, p < 0.001, 95% CI: 1.48, 1.99) as compared to those with less than 4 ANC contacts. About 26% of the respondents had any exposure to an ASHA worker. After controlling for ANC contacts, these respondents had three times the odds of institutional delivery (OR = 3.04, *p* < 0.001, 95% CI: 2.37, 3.89) compared to those who had no exposure to ASHA workers. Further, several sociodemographic variables were associated with institutional delivery. While age of spouse, age at marriage, level of education and urban residence were positively associated with institutional delivery; age of respondent and number of children were inversely associated with institutional delivery.

**Conclusions:**

Both frequency of ANC contacts and exposure to ASHA worker independently emerge as important determinants of institutional delivery. Furthermore, ASHA workers may have a crucial role in promoting antenatal care, thereby strengthening the association between ANC contacts and institutional delivery.

## Background

Trends in maternal health outcomes show that 99% of maternal deaths worldwide take place in developing countries [1]. Each day about 830 women die due to complications of pregnancy and child birth; and maternal mortality rate emerges as a key indicator of disparities between developed and developing countries [2]. In 2017, India recorded approximately 35,000 maternal deaths, and along with Nigeria accounted for over one-third of estimated global maternal deaths [3].

Quality care during pregnancy and through child birth is associated with improved maternal and child health outcomes [4, 5]. Evidence shows that delivery at a health facility assisted by skilled birth attendants and with access to emergency obstetric care is crucial to reducing maternal deaths [6, 7]. While the implementation of government programs aimed at improving maternal and child health outcomes have been associated with increased utilization of antenatal care and in-facility births in India [8]; there still exist considerable socio-economic inequities in access to institutional delivery services [9, 10].

### Determinants of institutional delivery

Previous research documents that socio-demographic characteristics such as household wealth, maternal education, age at marriage, caste and religion are important determinants of institutional delivery [11–16]. Apart from these, studies also found that knowledge or awareness regarding complications during pregnancy, cultural norms and beliefs, access to a health facility, availability of medicines, quality care, support from providers, perceived health benefits for mother and child, and antenatal care were some additional reasons shaping the decision on choice of place of delivery [11].

While studies in India have documented the association between timing and frequency of ANC contacts and improved maternal and child health outcomes [17, 18], there is limited evidence on frequency of ANC contacts as a determinant of institutional delivery [19, 20]. The 2016 WHO ANC model recommends a minimum of eight ANC contacts aimed at improving maternal, fetal and newborn health outcomes. This recommendation replaces the previous four-visit focused ANC model [21]. Pregnant women in developed nations are recommended to make nearly twice that many visits to a health care professional during pregnancy - once a month during the first 6 months of pregnancy, twice a month during the 7th and 8th month and weekly contacts after the 8th month until delivey [22]. Further, evidence from the South Asian region show that women adhering to the recent WHO recommendation of eight or more ANC contacts were more likely to receive all components of ANC services [23]. However, it is still unclear how many women in developing countries have 8 or more ANC contacts, and if increasing the number of ANC contacts significantly improves institutional delivery. In this study, we attempt to contribute to this body of evidence, and examined the proportion of women who attained at least eight ANC contacts and the effect of these contacts on institutional delivery.

### Role of ASHA worker

Aligned with the goal of reducing maternal and child deaths through improved access to maternal health services, the National Rural Health Mission (NRHM) was introduced in India in 2005 [24]. Crucial to the NRHM is the Accredited Social Health Activist (ASHA) who is tasked with the role of mobilizing, counselling and supporting pregnant women in the community, particularly in relation to institutional delivery, antenatal care and postnatal care [24]. The program was first launched in 2006 in 18 high focus states (Central, North and Western region - Bihar, Jharkhand, Madhya Pradesh, Chhattisgarh, Himachal Pradesh, Jammu and Kashmir, Uttar Pradesh, Uttaranchal, Orissa, and Rajasthan; Northeastern region - Arunachal Pradesh, Manipur, Assam, Nagaland, Meghlaya, Tripura, Mizoram, and Sikkim); and as of 2015, there are 859,331 ASHAs trained and working across the country. Although ASHA workers are primarily volunteers, they receive performace based compensation for facilitating maternal health service utilization through registering pregnant women, and enabling and motivating women to access antenatal care, postnatal care and institutional delivery [25]. However, despite the widespread size and scope of the ASHA program, few studies have used national level data to examine the effect of exposure to ASHA worker and the specific impact this contact has on institutional delivery [26, 27].

In this study, we ask the following questions:

1. Do women with more ANC contacts have a higher likelihood of institutional delivery controlling for socio-demographic factors?
2. Does exposure to ASHA worker increase the likelihood of institutional delivery, controlling for frequency of ANC contacts and socio-demographic factors?

## Methods

### Dataset

We used data from the India Human Development Survey-II (IHDSII), 2011–12 conducted by researchers from the University of Maryland and the National Council of Applied Economic Research, New Delhi. This survey is a nationally representative, multi-topic survey of 41,554 households and a sample of 39,523 women spread over 1503 villages and 971 urban neighborhoods across 33 states and union territories of India [28]. Household interviews were conducted with 33,510 ever-married women aged 15–49 years and included information regarding all births between 2000 and the interview date. We extracted data (*N* = 11,648) on respondents who reported having at least one birth since 2005. Women respondents were excluded from analyses if they had missing information on place of delivery (2733), ANC contacts (1861), or for socio-demographic variables. We further excluded 65 respondents who reported place of delivery as other than home or health facility. The final analytic sample consisted of 8711 respondents.

The study was reviewed by the Institutional Review Board at Boston College and considered exempt.

### Measures

We used a dichotomous outcome variable, *Institutional Delivery*, based on choice for place of delivery. Respondents who delivered at a health facility (including both public and private) were coded as 1, and those who responded that they delivered at home were coded as 0.

To measure *Frequency of ANC contacts*, we recoded the variable based on the question “How many antenatal care checkups have you had during your last pregnancy?”. This variable included three categories: Less than 4 contacts; 4–7 contacts; 8 or more contacts.

*Exposure to ASHA worker* was coded as a binary variable; respondents who had any exposure to an ASHA worker were coded as 1, otherwise as 0. Exposure to ASHA worker was defined based on whether respondent reported yes or reported being assisted by ASHA to any of the following questions [26]: “Where did you get a pregnancy card made?”; “Did you get help from anyone for making a pregnancy card/registration?”; “Who visited you when you were pregnant?”; “Who facilitated or motivated you to go to a health facility for delivery?”; and “Who arranged the transportation to take you to the health facility for delivery?” (see Table 1).

**Table 1:** List of variables used in analysis

Study variables	IHDS response categories	Recoded response categories
**Dependent variable**		
*Institutional Delivery* “What was place of delivery for your last pregnancy?”	This variable included three categories: Government hospital, private nursing home, home. ‘Home’ used as reference category.	0 = Home1 = Health facility
**Independent variables**		
*Number of antenatal care contacts* “How many antenatal care checkups have you had during your last pregnancy?”	Number/Don’t know ‘Less than 4 contacts’ used as reference category.	0 = Less than 4 contacts1 = 4–7 contacts2 = 8 or more contacts
*Exposure to ASHA worker*	Based on whether the respondent answered yes/ ASHA worker to any of these questions: “Where did you get a pregnancy card made?”; “Did you get help from anyone for making a pregnancy card/registration?”; “Who visited you when you were pregnant?”; “Who facilitated or motivated you to go to a health facility for delivery?”; “Who arranged the transportation to take you to the health facility for delivery?”.	0 = No1 = Yes
**Control variables**		
*Respondent’s Education*	This variable included five categories: no education, primary education (pre-primary to the completion of 5th grade of schooling), secondary education (6th grade to the completion of 10th grade); Higher secondary and above higher secondary (beyond 10th grade). ‘No education’ was used as the reference category.	0 = no education1 = primary education 2 = secondary education 3 = Higher secondary 4 = Above higher secondary (beyond 10th grade).
*Spouse’s education*	Same as above	
*Caste*	This variable included five categories: Forward caste (brahmins); Forward castes (excluding brahmins); Other Backward Classes; Scheduled Castes and Schedules Tribes. ‘Forward caste (Brahmin)’ was used as the reference category.	No recoding
*Religion*	This variable included seven categories: Hindu, Muslim, Christian, Sikh, Buddhist, Jain and Tribals. ‘Hindu’ was used as the reference category.	0 = Hindu1 = Muslim2 = Other religions
*Place of residence*	This variable included two categories: Rural, Urban ‘Rural’ was used as reference category.	No recoding
*Age at marriage*	Continuous variable representing woman’s age at marriage. ‘Below 18 years’ was used a reference category.	0 = Below 18 years 1 = 18 years and above
*Respondent’s age*	Continuous variable representing respondent’s age.	No recoding
*Spouse’s age*	Continuous variable representing spouse’s age.	No recoding
*Number of children*	Continuous variable representing number of children born.	No recoding
*Annual Household Wage*	Continuous variable representing annual household wage based on the sum of wage and salary incomes across all jobs of all individuals in the household.	No recoding

For details on the operationalization of all variables including the socio-demographic control variables, please see Table 1.

### Statistical analyses

We conducted weighted (probability weights) descriptive statistics, bivariate analyses, and logistic regression analyses to assess factors associated with institutional delivery and effect of exposure to ASHA worker on institutional delivery. Bivariate analysis was used to identify the association between all predictor variables used in the current study and the outcome variable. We then ran two separate logistic regression models. In model I, we examined associations between Frequency of ANC contacts and Institutional Delivery after controlling for socio-demographic characteristics (respondent’s age, spouse’s age, respondent’s education, spouse’s education, age at marriage, number of children, caste, religion, annual household wage, place of residence). In Model II, we added the Exposure to ASHA worker variable, and examined the effect of exposure to ASHA worker on institutional delivery, controlling for Frequency of ANC contacts and socio-demographic factors.

Data analyses were conducted using the statistical package Stata SE version 14.2 (Stata Corp, College Station, TX). In order to account for the complex sample design, survey weights were used to obtain representative estimates. Approximately 21% of cases had missing values, however we did not use multiple imputation as a technique to handle the missing cases due to large sample size and systematic nature of missing values. Respondents with missing values were significantly less likely to have institutional delivery (OR:0.56; CI: 0.52,0.60).

### Diagnostic tests

Prior to running the logistic regression models, we reviewed issues of multi-collinearity among the independent variables and also checked for goodness of fit. The mean highest variance inflation factor (VIF) was 2.32, which indicated lack of any serious problem of multi-collinearity [29].

## Results

Table 2 presents the socio-demographic profile of the respondents. Majority of the respondents were rural, had no formal education, and 40% were married before 18 years of age. The median annual household wage was 54,000 rupees (approx. 1211 USD). Majority of the sample was Hindu, and more than half of the sample belonged to lower caste groups. About 73% of sample had no exposure to an ASHA worker. In terms of ANC contacts, about half of the respondents had less than 4 ANC contacts during pregnancy and only about 15% had 8 contacts or more contacts. Approximately 27% of the respondents reported that they delivered at home. Of these, 20% had any exposure to an ASHA worker and 4% had eight or more ANC contacts. We also ran a Chi-Square Test of Independence for variables measuring frequency of ANC contacts and exposure to ASHA worker. We found that frequency of ANC contacts and exposure to ASHA worker are negatively, significantly correlated (χ^2^ = 119.84, *p* < 0.001).

**Table 2:** Weighted descriptive statistics results using IHDS-II from India (N = 8711)

Characteristics of Women and their Households (%, Mean, Standard Deviation)	Percentage (%) N = 8711
**Place of delivery (%)**	
Home	26.66
Health facility	73.34
**Frequency of ANC contacts (%)**	
Less than 4 visits	49.34
4–7 visits	36.02
8 or more visits	14.64
**Exposure to ASHA worker (%)**	
No	73.48
Yes	26.52
**Respondent’s Education (%)**	
No education	30.00
Primary	15.69
Secondary	37.12
Higher secondary	9.14
Above higher secondary	8.05
**Spouse’s Education (%)**	
No education	17.50
Primary	17.56
Secondary	43.13
Higher secondary	9.72
Above higher secondary	12.09
**Caste (%)**	
Forward caste	3.57
Forward/General (except Brahmin)	19.14
Other Backward Classes	39.81
Scheduled Castes	27.89
Scheduled Tribes	9.59
**Religion (%)**	
Hindu	81.64
Muslim	13.64
Other	4.73
**Place of residence (%)**	
Rural	68.75
Urban	31.25
**Age at marriage (%)**	
Below 18 years	39.19
18 years and above	60.81
**Mean age of respondent (Standard Deviation)**	27.79 (5.43)
**Mean age of spouse (Standard Deviation)**	32.63 (6.36)
**Mean number of children**	2.37 (1.49)
**Median Annual Household wage in rupees (IQR)**	54,000 (74960)

### Logistic regression analyses

Bivariate analysis indicated that all predictor variables used in the current study were significantly associated with the outcome variable. To examine factors influencing institutional delivery, two multiple logistic regression analyses were performed. The results of model I show a positive relationship between the frequency of ANC contacts and institutional delivery. The odds of institutional delivery among respondents who had 8 or more ANC contacts were about 3.39 times the odds for those who had less than 4 ANC contacts. Among the control variables, older respondents, those with higher education, those married at 18 years or above, living in households with higher annual household wage and respondents living in urban areas had higher odds of institutional delivery (See Table 3, Model I). In Model I, we found that compared to forward caste (Brahmin), respondents belonging to Other Backward Classes (OBC) had increased odds of institutional delivery. However, predicted odds of Muslim respondents having institutional delivery was 33% lower compared to their Hindu counterparts (OR = 0.67, *p* < 0.05, 95% CI: 0.46, 0.97). Further, higher number of children was associated with decreased odds of institutional delivery (OR = 0.82, *p* < 0.001, 95% CI: 0.76, 0.87).

**Table 3:** Logistic regression analyses demonstrating influence of ASHA workers on institutional delivery in India (N = 8711)

Variables	Model 1	95% CI	Model 2	95% CI
	Odds ratio		Odds ratio	
			(No exposure = 0; Exposure to ASHA worker = 1)	
**Respondent’s Education** *(Reference: No education)*				
Primary	1.04	(0.78, 1.38)	1.04	(0.79, 1.38)
Secondary	1.82***	(1.39, 2.40)	1.83***	(1.40, 2.38)
Higher secondary	2.17**	(1.33, 3.55)	2.20**	(1.36, 3.58)
Above higher secondary	3.03***	(1.72, 5.35)	3.23***	(1.89, 5.52)
**Spouse’s Education***(Reference: No education)*				
Primary	0.97	(0.75, 1.26)	0.98	(0.75, 1.29)
Secondary	1.08	(0.86. 1.35)	1.10	(0.88. 1.38)
Higher secondary	1.11	(0.76, 1.63)	1.06	(0.73, 1.53)
Above higher secondary	1.43*	(1.01, 2.01)	1.48*	(1.05, 2.08)
**Caste***(Reference: Brahmin)*				
Forward/General (except Brahmin)	1.51	(0.92, 2.46)	1.49	(0.94, 2.38)
Other Backward Classes	1.49*	(1.04, 2.14)	1.47*	(1.04, 2.08)
Scheduled Castes	1.38	(0.94, 2.05)	1.32	(0.90, 1.93)
Scheduled Tribes	0.97	(0.69, 1.37)	0.88	(0.64, 1.22)
**Religion** *(Reference: Hindu)*				
Muslim	0.67*	(0.46, 0.97)	0.64*	(0.45, 0.92)
Other	0.78	(0.49, 1.23)	0.88	(0.56, 1.38)
**Place of residence** *(Reference: Rural)*				
Urban	2.00***	(1.58, 2.52)	2.45***	(1.91, 3.14)
**Age at marriage** *(Reference: Less than 18 years)*				
18 years and above	1.26**	(1.04, 1.54)	1.26*	(1.03, 1.54)
**Respondent’s Age**	0.95**	(0.92, 0.98)	0.95**	(0.92, 0.98)
**Spouse’s Age**	1.04**	(1.01, 1.07)	1.04**	(1.01, 1.06)
**Number of children**	0.82***	(0.76, 0.87)	0.80***	(0.75, 0.86)
**Annual Household Wage**	1.00***	(1.00, 1.00)	1.00***	(1.00, 1.00)
**Frequency of antenatal contacts***(Reference: Less than 4 contacts)*				
4–7 visits	1.72***	(1.48, 1.99)	1.84***	(1.57, 2.15)
8 or more visits	3.39***	(2.26, 5.08)	3.93***	(2.66, 5.81)
**Exposure to ASHA worker***(Reference: No)***Yes**				
			3.04***	(2.37, 3.89)
**Constant**	1.36	(0.74, 2.48)	0.86	(0.47, 1.57)
**Observations**	8711		8711	
**Wald** χ^2 a^	1611.38***		1895.65***	

* *p* < 0.05; ** *p* < 0.01; *** *p* < 0.001; ^a^Computed using unweighted data

Within Model II we added the variable, exposure to ASHA worker to Model I. We found that respondents who were exposed to ASHA worker had 3.04 (p < 0.001, 95% CI: 2.37, 3.89) times the odds of having institutional delivery in comparison with those who had no exposure to ASHA worker. Further, the strength of relationship between frequency of ANC contacts and institutional delivery improved in Model II (8 or more contacts: OR changed from 3.39 in Model I to 3.93 in Model II; 4–7 contacts: OR changed from 1.72 in Model I to 1.84 in Model II).

## Discussion

Globally, studies indicate that institutional delivery has led to improved maternal and child health outcomes [4–7]. Evidence from India shows that introduction of government programs focused on incentivizing delivery at health facility has been associated with increases in in-facility births and significant reductions in perinatal and neonatal mortality [8]. Within this context, our study explores factors that influence institutional delivery and the role of the ASHA worker. Previous research documents importance of several individual and system level factors as determinants of institutional delivery [11–16, 30]. Contributing to this body of evidence, in the discussion that follows, we highlight some of the main findings.

First, we found that frequency of ANC contacts is a crucial determinant of institutional delivery. More specifically, increasing ANC contacts to eight or more was significantly associated with increased odds of institutional delivery. Antenatal care has long been regarded as a core component of routine maternal and child health services and as a strategy to reduce maternal and neonatal mortalities [21]. Evidence from India and other countries in South Asia show that antenatal care is associated with improved maternal and child health outcomes [18–20, 23, 31]. In particular, studies demonstrate that increased frequency of ANC contacts are associated with reduced risk of neonatal mortality, and increased odds of institutional delivery [18–20]. The reason for this may be that apart from routine check-ups, women who have had higher number of ANC contacts would also be more likely to receive information regarding benefits of delivery at a health facility and knowledge about danger signs and obstetric complications. Most pregnant women in developed nations including the United States are recommended to make far more than eight visits during pregnancy; women in developed countries may make 13 to 14 visits to a health care professional before they deliver [22]. Developing countries considered prenatal visits more seriously only since the United Nations conference on Safe Motherhood Initiative held in Nairobi, Kenya in 1987. This conference drew attention to the fact that over half a million women were dying from preventable maternity complications and recommended member countries to reduce maternal mortality by 50% by 2000. It called for specific actions: a pregnant woman without any complications should make at least 4 visits to a skilled health attendant and more visits as needed if the pregnancy is complicated; and that delivery be done at a health facility attended by skilled health personnel [32]. More recently, studies comparing the reduced visit ANC model (at least 4 contacts) with the recent standard (at least 8 contacts) model found that standard model with eight or more contacts was associated with better maternal, fetal and neonatal outcomes [33, 34]. Additionally, evidence shows that women from high, medium and low resource settings prefer more contact with antenatal services and do not like reduced visit schedules; particularly, valuing the opportunity to build supportive relationships during their pregnancy [35]. Based on this evidence, the WHO recommends eight or more ANC contacts as crucial to reducing perinatal death [21]. A 2018 study in Bangladesh found a significant positive association between eight or more ANC contacts and receipt of increased number of ANC services [23]. While studies in India have examined the association between frequency of ANC contacts and maternal and child health outcomes [18–20], none of these studies have focused on the effect of eight or more contacts. Although the current guidelines in India recommend a minimum of 4 or more contacts [36], our results highlight the importance of the recent WHO recommendation of eight or more ANC contacts, and of the link between frequency of ANC contacts and institutional delivery. Also, to reduce global health disparities in maternal deaths, going forward, it makes sense to aim even higher and encourage women in developing countries like India to increase the number of ANC contacts to eight or more.

Second, ASHA workers are crucial for increasing institutional delivery. While we found that exposure to ASHA worker is associated with increased odds of institutional delivery, addition of ASHA worker variable into the model also increased effect of ANC contacts. Community health workers are key to bridging the gap between health services and utilization, and there is a global need to increase numbers of community health workers and to integrate them directly into health systems [37]. In India, ASHA workers are the cornerstone of the National Rural Health Mission, and are crucial to counselling and mobilizing women to access reproductive health services. Evidence shows that presence of ASHA workers is associated with increased frequency of ANC contacts, increase in skilled birth attendance and facility births, decreased unmet need for family planning and increase in immunization coverage [26, 27, 30]. Qualitative studies indicate that the ASHA workers played an important role in health promotion, awareness generation and counseling on safe delivery, as well as facilitating access to health care services through arrangement of transportation to the hospital [38]. Overall, while studies suggest a positive influence of ASHA workers on maternal and child health outcomes, there is some conflicting evidence on effectiveness of the ASHA program in relation to institutional delivery. A recent study using national level data from India showed that increased placement of ASHA workers had no direct impact on institutional delivery, but was associated with increase in other aspects of healthcare utilization [27]. In contrast, another study found that receipt of ASHA services was associated with increase in maternal health service utilization including antenatal care and institutional delivery [26]. Our study results lend support to this body of evidence and demonstrates that exposure to ASHA worker is associated with increased odds of institutional delivery, controlling for frequency of ANC contacts and socio-demographic factors. Further, we found that the strength of association between frequency of ANC contacts and institutional delivery increased in Model II where we included the variable on exposure to ASHA worker. As previous studies have found a negative correlation between ASHA workers and ANC contacts [26], we suspected a suppression effect of ASHA worker variable. Several authors have called a variable a suppressor when a suppressor and another predictor are positively correlated with the outcome variable but are negatively correlated with each other [39]. Using the Chi-Square Test of Independence, we found that the variables measuring frequency of ANC contacts and exposure to ASHA worker are negatively, significantly correlated, indicating that ASHA workers served as a suppressor, suppressing outcome-irrelevant variance in ANC contacts resulting in its increase in regression weight [40, 41]. These findings contribute to the body of evidence demonstrating the positive effect of ASHA worker on service utilization, and additionally highlight their potential role in awareness generation and as a motivating factor to access antenatal care. Despite the positive effect of ASHA workers on maternal and child health outcomes, there is considerable regional variation in reported receipt of ASHA services [26] and our study results show that approximately three-fourths of the sample reported no exposure to an ASHA worker. Future research should focus on understanding the reasons why the ASHA workers have been unable to reach certain sections of the population and what strategies may be used to increase accessibility.

Third, consistent with previous studies, our study results found that socio-demographic factors such as women’s age, spouse’s age, women’s age at marriage, number of children, education, caste, religion and annual household wage were significantly associated with institutional delivery. Education emerges as a strong predictor of institutional delivery in previous research [11, 14] and within our study we found that women who had primary or secondary level education had almost twice the odds of institutional delivery as compared with those who reported having no education. It is likely that educated women would have greater awareness about maternal health care services and the utilization of these services [42], greater ability to afford health care, and freedom to make health-related decisions [43]. Furthermore, our study results also lend support to prior research which shows that husband’s education level and knowledge about pregnancy-related care emerge as a crucial determinant of utilization of maternal health services [44, 45]. Within our study, we recoded age at marriage into a dichotomous variable and our results indicate that women married after 18 years of age are 26% more likely to have an institutional delivery. Evidence shows that age at marriage influences reproductive health outcomes [46] and maternal health service utilization [47]; with risks of stillbirth, miscarriage, and complications during pregnancy being more pronounced among women married below 14 years of age [46]. While the government has implemented programs to increase age at marriage, there is a need for sustained efforts towards increasing knowledge, awareness and access to healthcare among married adolescents. Additionally, compared to Hindus, Muslim women were less likely to have institutional delivery; in contrast, compared with those belonging to forward caste (Brahmin), women who belong to Other Backward Classes (OBC) were more likely to have an institutional delivery. These social conditions vary between states and within different communities in each state. Future research should focus on addressing the underlying reasons of religion and caste based inequities in utilization of maternal health services, in addition to state-level policy formulation that is tailored to specific contexts [42].

This study has some limitations. The chosen study population only includes women who had at least one birth post 2005, and thus excludes those who gave birth prior to 2005 and their health behavior. This study does not allow us to capture the influence of cultural norms and beliefs as determinants of institutional delivery. Further, we were unable to account for travel time and distance to health facility within our model, which may influence decisions regarding choice of place of delivery. Lastly, we were unable to control for Janini Suraksha Yojana (JSY) beneficiaries within the model and thus cannot separate the effect of visit by ASHA worker and impact of conditional cash transfer through JSY scheme. Despite these limitations, our study makes an important contribution by using national level data to examine the determinants of institutional delivery. This study calls attention to the importance of promoting eight or more ANC contacts among pregnant women in India as a key strategy to improve maternal health outcomes. Also, we find that the role ASHA worker in improving maternal health is further validated. We have utilized the 2012 IHDS data as it contains the required variables on ANC contacts and exposure to ASHA worker.

## Conclusion

Our study highlights frequency of ANC contacts as a key determinant of institutional delivery, thus underscoring the need for strengthening and improving antenatal care services. Furthermore, our results show that apart from having a direct influence on institutional delivery, ASHA workers may also play a role in increasing institutional delivery by promoting uptake of antenatal care services. While ASHA workers are incentivized for promoting institutional delivery, government programs should also focus on the role of ASHA workers in promoting antenatal care. Further research is required to identify effective strategies for training and strengthening the ASHA workforce, as a crucial step to bridging the gap between the community and the health system.

## Data Availability

The datasets generated and/or analyzed during the current study are available in the Data Sharing for Demographic Research (DSDR) repository, https://www.icpsr.umich.edu/web/DSDR/studies/36151/datadocumentation#.

## Abbreviations

NRHM: National Rural Health Mission
ASHA: Accredited Social Health Activist
ANC: Antenatal Care
WHO: World Health Organization
JSY: Janini Suraksha Yojana
